# Epigenetic alterations in skin homing CD4^+^CLA^+^ T cells of atopic dermatitis patients

**DOI:** 10.1038/s41598-020-74798-z

**Published:** 2020-10-22

**Authors:** Nathalie Acevedo, Rui Benfeitas, Shintaro Katayama, Sören Bruhn, Anna Andersson, Gustav Wikberg, Lena Lundeberg, Jessica M. Lindvall, Dario Greco, Juha Kere, Cilla Söderhäll, Annika Scheynius

**Affiliations:** 1 Department of Clinical Science and Education, Karolinska Institutet, and Sachs’ Children and Youth Hospital, Södersjukhuset, 118 83 Stockholm, Sweden; 2 National Bioinformatics Infrastructure Sweden (NBIS), Science for Life Laboratory, Department of Biochemistry and Biophysics, Stockholm University, 10691 Stockholm, Sweden; 3 Department of Biosciences and Nutrition, Karolinska Institutet, Stockholm, Sweden; 4 Department of Medicine Solna, Translational Immunology Unit, Karolinska Institutet, Stockholm, Sweden; 5 Dermatology and Venereology Unit, Karolinska University Hospital, Stockholm, Sweden; 6 Faculty of Medicine and Health Technology, Tampere University, Tampere, Finland; 7 Institute of Biosciences and Medical Technologies (BioMediTech), Tampere, University, Tampere, Finland; 8 Institute of Biotechnology, University of Helsinki, Helsinki, Finland; 9 Department of Women’s and Children’s Health, Karolinska Institutet, Stockholm, Sweden; 10 Science for Life Laboratory, Karolinska Institutet, Stockholm, Sweden; 11 Present Address: Institute for Immunological Research, University of Cartagena, Cartagena, Colombia

**Keywords:** Immunology, Adaptive immunity, Cytokines, Gene regulation in immune cells, Inflammation, Lymphocytes, Immunological disorders, Skin diseases, Epigenetics, Genome, Gene ontology, Gene regulatory networks, Microarrays

## Abstract

T cells expressing the cutaneous lymphocyte antigen (CLA) mediate pathogenic inflammation in atopic dermatitis (AD). The molecular alterations contributing to their dysregulation remain unclear. With the aim to elucidate putative altered pathways in AD we profiled DNA methylation levels and miRNA expression in sorted T cell populations (CD4^+^, CD4^+^CD45RA^+^ naïve, CD4^+^CLA^+^, and CD8^+^) from adult AD patients and healthy controls (HC). Skin homing CD4^+^CLA^+^ T cells from AD patients showed significant differences in DNA methylation in 40 genes compared to HC (*p* < 0.05). Reduced DNA methylation levels in the upstream region of the interleukin-13 gene (*IL13*) in CD4^+^CLA^+^ T cells from AD patients correlated with increased *IL13* mRNA expression in these cells. Sixteen miRNAs showed differential expression in CD4^+^CLA^+^ T cells from AD patients targeting genes in 202 biological processes (*p* < 0.05). An integrated network analysis of miRNAs and CpG sites identified two communities of strongly interconnected regulatory elements with strong antagonistic behaviours that recapitulated the differences between AD patients and HC. Functional analysis of the genes linked to these communities revealed their association with key cytokine signaling pathways, MAP kinase signaling and protein ubiquitination. Our findings support that epigenetic mechanisms play a role in the pathogenesis of AD by affecting inflammatory signaling molecules in skin homing CD4^+^CLA^+^ T cells and uncover putative molecules participating in AD pathways.

## Introduction

Atopic dermatitis (AD) is a common chronic inflammatory skin disorder characterized by intense pruritus and xerosis that usually concur with allergic sensitization and elevated plasma immunoglobulin E (IgE) levels^1^. In recent years, epigenetic modifications are being recognized as key players in the alterations leading to complex inflammatory diseases including AD^2–7^. By affecting DNA methylation and non-coding RNA levels, these epigenetic modifications determine a complex network of chemical signatures that regulate mRNA expression and are heritable to daughter cells. Therefore, they control the activation or silencing of genes that are critical for cell memory and homeostasis, by regulating which genes are expressed, when, and where^8^. DNA methylation is an epigenetic modification involving the addition of a methyl group in a cytosine preceding a guanine (CpG site); usually when gene promoters are methylated the gene transcription is suppressed while reduced methylation levels activate genes and promote their expression. When CpG sites have significant differences in DNA methylation between patients and controls, they are named differentially methylated probes (DMPs), and if many of them are close to each other in a gene it is defined as a differentially methylated region (DRM). The contribution of this epigenetic modification in AD has been evaluated in diverse tissues, for instance, DNA methylation in the interleukin 4 receptor gene (*IL4R*) as detected in cord blood samples was associated with AD at age 12 months^9^. Decreased methylation in the thymic stromal lymphopoietin (*TSLP*) promoter resulting in TSLP overexpression has been detected in lesional skin biopsies of AD patients^10^. DNA methylation differences have also been detected in the epidermis of AD patients^11^. Altered expression of microRNAs has also shown to play a crucial role in type 2 immunity and in promoting pathogenic mechanisms in AD^12–15^. However, very few studies have analyzed these epigenetic signatures in purified immune cells from AD patients^3^.

Circulating T cells can infiltrate human skin as a mechanism of immunosurveillance. They are identified by the expression of the cutaneous lymphocyte-associated antigen (CLA), a cell surface glycoprotein that interacts with E-selectin on the endothelium of postcapillary venules and allows their selective transmigration from the peripheral circulation to the dermis^16^. AD patients have a higher percentage of circulating CLA^+^ T cells compared to HC^17,18^ and the selective expansion of CLA^+^ T cells in severe AD involves populations with Th2 and Th22 profiles^19^. Indeed, the frequencies of CLA^+^ Th2 T cells were similarly expanded across all AD age groups compared with control subjects^20,21^. CLA^+^ T cells in the peripheral blood of patients with AD are also functionally disturbed, with increased expression of cytokine receptors that promote their Th2-skewed pattern^19^. Moreover, they induce IgE production in B cells, enhance eosinophil survival^22^ and display features of in vivo activation such as the spontaneous release of IL-13 and increased levels of CD25, CD40L and HLA-DR^23^. Circulating CD4^+^CLA^+^ T cells in patients with acute AD furthermore produce significantly higher levels of IL-4, IL-13 and tumor necrosis alpha compared to patients with chronic AD or healthy controls (HC)^24^. After skin homing, the CLA^+^ T cells form dermal infiltrates, secrete interferon gamma and become protected from activation-induced cell death^25^. Recent studies indicate that circulating CLA^+^ T cells share phenotype, function, and clonotypes with tissue resident CLA^+^ T cells in the skin^26^ in agreement with that blood CD4^+^CLA^+^ T cells are a reliable surrogate marker of the inflammatory events occurring in the skin^16,27^. It has also been observed that interaction with external signals from allergens or the skin microbiome can activate CLA^+^ T cells and promote their pro-inflammatory state^28^. Indeed, CLA^+^ T cells are memory cells and antigen-experienced, so they can recognize epitopes in airborne and food allergens^29–32^ as well as in *Staphylococcus aureus*^18,33^.

Even though the alterations in CLA^+^ T cells from AD patients are well documented^34^ their extensive molecular characterization, needed to clarify the pathogenesis of AD, is currently missing. Here we compared genome-wide DNA methylation levels and miRNA expression in four sorted blood lymphocyte populations (CD4^+^, CD4^+^CD45RA^+^ naïve, CD4^+^CLA^+^ and CD8^+^) isolated from AD patients and HC. This study shows for the first time that skin-homing CD4^+^CLA^+^ memory T cells from AD patients contain dysregulated epigenetic signatures, including differences in DNA methylation in 40 protein-coding genes and 16 differentially expressed miRNAs. An analysis integrating the coordinated response in miRNAs and CpG probes revealed two groups with strong antagonistic behaviors that recapitulated the differences between AD patients and HC. Functional analysis of the genes linked to these groups revealed their association with key cytokine signaling pathways and protein ubiquitination. Our findings support that epigenetic mechanisms play a role in the pathogenesis of AD by affecting inflammatory signaling molecules in skin-homing CD4^+^CLA^+^ memory T cells and reveal novel disease candidates.

## Results

### Purity of isolated T cell populations

Based on our previous observations that sorting by cell-type is crucial for the interpretation of results derived from DNA methylation studies^35,36^, we isolated four populations of peripheral blood lymphocytes (CD4^+^, CD4^+^CD45RA^+^ naïve, CD4^+^CLA^+^ and CD8^+^ from AD patients with severe phenotype and HC (Table 1). The mean cell surface expression of CD4 and CD8 markers was over 90% in the respective sorted fractions (Supplementary Table S1 online). The mean CD45RA expression was 90% in the sorted naïve cells. The CD45RO^+^ marker was expressed on 85% of the sorted CD4^+^CLA^+^ lymphocytes, confirming their phenotype as memory cells. After cell isolation, we found no significant differences in the mean proportions of sorted positive cells between AD patients and HC (*t*-test *p* > 0.05, Supplementary Table S1 online).

**Table 1:** Descriptive characteristics of the participants at 450 ml blood donation.

	Healthy controls	AD patients
Sample size (n)^a^	10	10
Age, years (average ± SD)	37 ± 13.9	34.9 ± 14.7
SCORAD (objective)^b^	n.a	44 ± 5.5
Total plasma IgE kU_A_/L, (median, IQR)^c^	16.5 (8.2 – 22.5)	1750 (725 – 3050)
Phadiatop ≥ 0.35 kU /L, (n)^d^	1/10	10/10
Asthma, yes (n)^e^	0/10	4/10
Rhinitis, yes (n)^e^	0/10	10/10

^a^Given the sample size and to avoid confounding effects by gender, only males were analyzed.

^b^SCORAD: SCORing Atopic Dermatitis. Moderate eczema (score 15 to 40); severe eczema (> 40); n.a = not applicable.

^c^Determined by ImmunoCAP (Thermo Fisher, Uppsala, Sweden). Reference value in this population is 122 kUA/L; IQR = interquartile range.

^d^Phadiatop = analyses of IgE antibodies in plasma to a mix of aeroallergens including birch, timothy, mugwort, mite, cat, dog, horse and mold (Thermo Fisher).

^e^The presence of physician-diagnosed asthma and/or rhinitis was obtained by questionnaire at the time of blood sampling.

### Comparative analysis of DNA methylation between AD patients and HC

**Figure 1:**
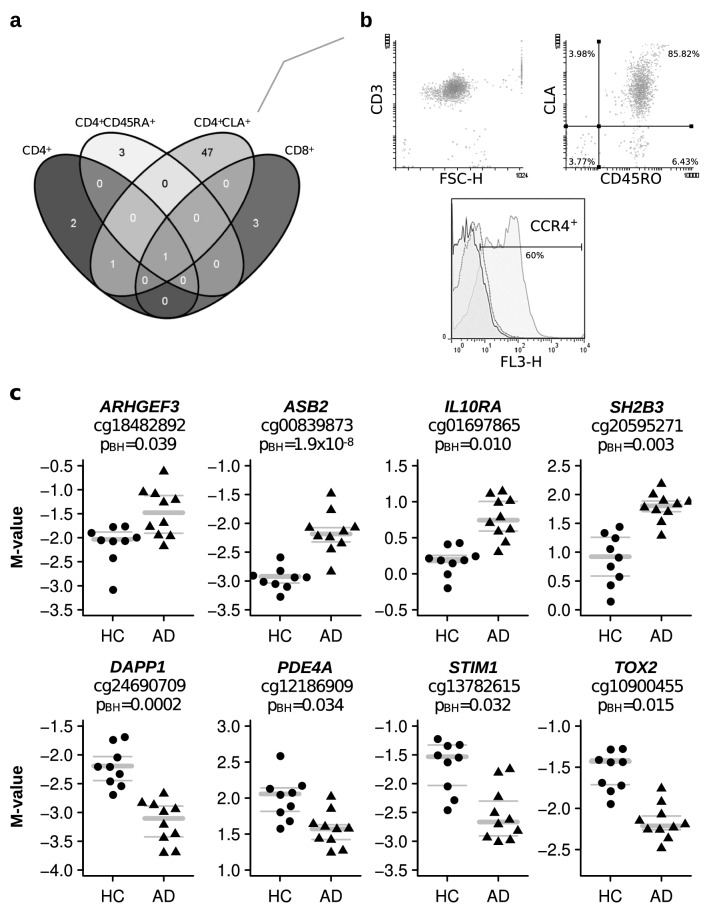
Differentially methylated probes (DMPs) in peripheral blood T cells between AD patients and HC. (**a**) Venn diagram showing the overlap of DMPs in four different sorted T cell populations. Plotted with the open webtool venny 2.0 (https://bioinfogp.cnb.csic.es/tools/venny/). (**b**) Representative flow cytometry analysis of CD3, CLA, CD45RO and CCR4 in sorted CD4^+^CLA^+^ T cells. Numbers within quadrants represent percentage of cells. FSC-H: forward scatter height; in histogram solid black line: unstained; dotted line: isotype control; gray line: anti CCR4 staining. (**c**) Eight DMPs in CD4^+^CLA^+^ T cells. DNA methylation levels are expressed as M-values, gray bars indicate mean (bold), upper and lower (thin) quartiles. M values above 1 represent that the CpG site is methylated, and M values below − 1 represent that the CpG site is demethylated. Each dot represents an individual, HC (n = 9) and AD patients (n = 10). P_BH_ = Benjamini Hochberg *p* value.

The analysis of the genome-wide DNA methylation levels in the four sorted T cell populations revealed that skin-homing CD4^+^CLA^+^ T cells were the subset with the largest numbers of differentially methylated CpG probes (DMPs, n = 49) between AD patients and HC (Fig. 1a). These cells expressed the CD3^+^ marker, as well as the CLA^+^ and the CD45RO^+^ markers, and in about 60% of the population the CCR4 receptor (Fig. 1b). The 49 DMPs in these circulating CD4^+^CLA^+^ T cells (BH corrected *p* value < 0.05) mapped to 35 genes (Table 2). Further analysis of DMRs revealed 5 genes with more than 3 DMPs in the CD4^+^CLA^+^ T cells of the AD patients compared to HC (Table 3). These results refined the signals detected in *GPR55*, *MAN1A1* and *CDHR3* by using the DMP analysis (Table 2) and uncovered methylation differences in the genes encoding the transcription factor estrogen receptor alpha (*ESR1*) and the nuclear receptor co-repressor 2 (*NCOR2*) (Table 3), suggesting that in AD patients the epigenome of circulating CD4^+^CLA^+^ T cells is affected in regions encoding transcription factors. The annotated list of 40 differentially methylated genes between AD patients and HC with their cell location and known function are presented in Table 4.

**Table 2:** Annotated list of the 49 differentially methylated probes (DMPs) in CD4^+^CLA^+^ cells of AD patients.

Illumina 450K id	chr	Position (strand)	Gene symbol	Relation to island	Enhancer	DHS	logfc	*p* value	Benjamini Hochberg *p* value
**Decreased DNA methylation in AD patients**									
cg06460587	chr6	31650930 (−)		Island		Yes	−1.38	3.75E−06	0.035
cg24531977	chr5	56204891 (+)	*C5orf35*	N_Shore			−1.14	2.46E−06	0.032
cg24690709	chr4	100768712 (+)	*DAPP1*	OpenSea			−1.03	6.73E−08	0.0029
cg26077005	chr6	27236793 (+)		OpenSea			−0.99	1.85E−07	0.0062
cg04853218	chr14	55769688 (+)	*FBXO34*	OpenSea	Yes		−0.94	1.01E−09	9.94E−05
cg08214808	chr11	45922166 (−)	*MAPK8IP1*	Island			−0.91	2.13E−06	0.031
cg11770323	chr13	80066032 (+)	*NDFIP2*	OpenSea	Yes		−0.90	4.18E−08	0.0020
cg21022949	chr2	231809697(+)		OpenSea			−0.88	5.38E−07	0.011
cg07182616	chr14	33409812 (+)	*NPAS3*	OpenSea			−0.87	3.34E−06	0.034
cg13782615	chr11	4079556 (+)	*STIM1*	OpenSea	Yes		−0.86	2.48E−06	0.032
cg00726151	chr14	97881274 (−)		OpenSea	Yes		−0.80	3.26E−11	6.50E−06
cg06854264	chr1	200861254 (+)	*C1orf106*	S_Shore		Yes	−0.76	3.23E−06	0.034
cg03207915	chr6	119669112 (+)	*MAN1A1*	N_Shore			−0.76	2.57E−06	0.032
cg01436550	chr16	24781512 (+)	*TNRC6A*	OpenSea	Yes		−0.75	3.42E−06	0.034
cg12741231	chr8	19321936 (−)	*CSGALNACT1*	S_Shelf			−0.73	1.14E−10	1.51E−05
cg07343739	chrX	46617524 (+)	*SLC9A7*	N_Shore	Yes		−0.72	2.37E−06	0.032
cg05313153	chr8	119122430 (+)	*EXT1*	N_Shore			−0.71	3.92E−07	0.0097
cg07633835	chr10	5938186 (−)	*FBXO18*	OpenSea	Yes		−0.70	3.48E−06	0.034
cg25360385	chr12	51786547 (−)	*GALNT6*	S_Shore			−0.70	3.05E−06	0.034
cg19722656	chr6	119669372 (−)	*MAN1A1*	N_Shore			−0.67	1.01E−06	0.018
cg26780915	chr7	105519144 (+)		S_Shore	Yes		−0.66	1.25E−09	9.94E−05
cg03405260	chr17	77786344 (+)		Island			−0.65	5.10E−06	0.043
cg10900455	chr20	42545099 (−)	*TOX2*	Island		Yes	−0.65	7.91E−07	0.015
cg08416875	chr6	119669226 (−)	*MAN1A1*	N_Shore			−0.64	4.10E−06	0.037
cg13607082	chr12	122652224 (−)	*LRRC43*	OpenSea			−0.64	3.59E−07	0.0095
cg15447017	chr1	156095882 (+)	*LMNA*	OpenSea			−0.63	3.49E−07	0.0095
cg12454975	chrX	103356930 (−)	*MCART6/ZCCHC18*	N_Shore		Yes	−0.63	9.12E−07	0.017
cg08062822	chrX	103356845 (−)	*MCART6/ZCCHC18*	N_Shore		Yes	−0.62	2.49E−06	0.032
cg12186909	chr19	10533016 (−)	*PDE4A*	S_Shore			−0.57	3.10E−06	0.034
cg12589298	chr19	50828905 (−)	*KCNC3*	Island			−0.57	2.74E−06	0.033
cg07910680	chr18	56296449 (+)	*ALPK2*	OpenSea	Yes		−0.55	5.41E−06	0.044
cg08494390	chr4	87980297 (−)	*AFF1*	OpenSea	Yes		−0.54	5.90E−06	0.047
cg03403880	chr2	157255372 (−)		N_Shore			−0.52	2.66E−07	0.0081
cg14523284	chr5	131993614 (−)	*IL13*	S_Shore			−0.49	3.94E−06	0.036
cg02712553	chr10	64136038 (−)	*ZNF365*	S_Shore			−0.49	1.90E−06	0.029
cg17347326	chr17	77779426 (+)		S_Shore	Yes		−0.46	3.39E−08	0.0019
cg05649724	chr14	102415204 (−)		Island			−0.45	4.61E−07	0.010
**Increased DNA methylation in AD patients**									
cg16312212	chr8	18941574 (+)		OpenSea	Yes		3.23	1.73E−06	0.027
cg00211609	chr1	1178039 (−)	*FAM132A*	Island			1.26	5.28E−09	0.00035
cg04871131	chr7	94954202 (+)	*PON1*	S_Shore	Yes		1.01	1.09E−06	0.018
cg20595271	chr12	111889200 (+)	*SH2B3*	OpenSea			0.85	8.71E−08	0.0034
cg00839873	chr14	94421989 (−)	*ASB2*	OpenSea			0.77	3.64E−06	0.035
cg18482892	chr3	56833426 (−)	*ARHGEF3*	N_Shelf			0.73	4.46E−06	0.039
cg01956154	chr14	94423399 (−)	*ASB2*	OpenSea	Yes		0.71	5.00E−14	1.99E−08
cg26800893	chr11	67184596 (−)	*ATPGD1*	S_Shore	Yes		0.68	1.89E−07	0.0062
cg08943180	chr1	244516022 (+)	*C1orf100*	OpenSea			0.59	5.15E−06	0.043
cg05523877	chr10	72185663 (+)	*EIF4EBP2*	OpenSea			0.53	1.53E−06	0.025
cg01697865	chr11	117856007 (+)	*IL10RA*	N_Shore			0.53	5.23E−07	0.011
cg21786381	chr11	75234078 (−)	*GDPD5*	N_Shelf			0.48	3.11E−06	0.034

chr = chromosome.

pos = genome coordinate (hg19); DHS = DNAse hypersensitivity site.

logfc = difference between AD patients and HC.

**Table 3:** Differentially methylated regions (DMRs) with decreased DNA methylation in CD4^+^CLA^+^ T cells from AD patients compared to HC.

Gene	Chr	DMR Start	Width^a^	CpGs	ID	logfc	*p* value	Benjamini–Hochberg * p* value
*GPR55*	2	231,790,037	776	4	cg16382047 cg14254999 cg19827923 cg13531460	−0.45	5.1 × 10^−7^	0.01
		231,809,610	87	3	cg08840017 cg25013095 **cg21022949**	−0.73	1.5 × 10^−8^	0.0006
*MAN1A1*	6	119,669,112	260	4	**cg03207915 cg08416875** cg02578070 **cg19722656**	−0.68	2.5 × 10^−8^	0.0008
*ESR1*	6	152,126,895	1910	23	See footnote^b^	−0.36	3.6 × 10^−6^	0.03
*CDHR3*	7	105,515,219	677	2	cg03619256 cg20186907	−0.55	7.8 × 10^−7^	0.01
		105,518,489	655	2	cg00494287 **cg26780915**	−0.68	3.6 × 10^−10^	2.4 × 10 − 5
*NCOR2*	12	124,876,433	217	3	cg16337430 cg16217368 cg11050793	−0.53	1.8 × 10^−6^	0.02

^a^DMR analysis is based on *minfi* to collapse connected DNA methylation probes by distance rules; width in base pairs.

^b^Sites in *ESR1* cg21157690, cg17264271, cg15543523, cg26089753, cg08884395, cg01715172, cg21608605, cg20627916, cg07671949, cg23164938, cg23165623, cg21614759, cg19411146, cg21950534, cg11813455, cg24900983, cg05171584, cg23467008, cg22839866, cg23009221, cg27316393, cg00655307, cg01777019.

CpG sites indicated in bold were also found as differentially methylated CpG sites in the DMP analysis (see Table 2). Chr: chromosome.

**Table 4:** A summary of the 40 differentially methylated genes in CD4^+^CLA^+^ T cells of AD patients compared to HC (including genes with DMPs and DMRs).

Gene symbol	Gene name	Cell location	Function
*AFF1*	AF4/FMR2 family, member 1	Nucleus	Transcription factor
*ALPK2*	Alpha-kinase 2	Nucleoplasm	Kinase recognize phosphorylated peptides
*ARHGEF3*	Rho guanine nucleotide exchange factor (GEF) 3	Cytosol	Guanine nucleotide exchange factor
*ASB2*	Ankyrin repeat and SOCS box containing 2	Cytosol?	Protein polyubiquitination/proteasome degradation
*ATPGD1 aka. CARNS1*	Carnosine synthase 1	Unknown	Synthesis of carnosine and homocarnosine
*C1orf100*	Chromosome 1 open reading frame 100	Unknown	Uncharacterized protein 147aa 17kda
*C1orf106*	Chromosome 1 open reading frame 106	Unknown	Uncharacterized protein 663aa 72.9kda
*C5orf35*	Chromosome 5 open reading frame 35	Unknown	Uncharacterized protein 147aa 17kda
*CDHR3*	Cadherin related family member 3	Plasma membrane	Calcium-dependent cell adhesion proteins
*CSGALNACT1*	Chondroitin sulfate N-acetylgalactosaminyltransferase 1	Golgi	Peptidoglycan glycosyltransferase activity
*DAPP1*	Dual adaptor of phosphotyrosine and 3-phosphoinositides	Plasma membrane, cytosol	PI3K signaling
*EIF4EBP2*	Eukaryotic translation initiation factor 4E binding protein 2	Unknown	Bind eIF4E and inhibit translation initiation
*ESR1*	Estrogen receptor 1	Plasma membrane, cytosol and nucleus	Ligand-activated transcription factor
*EXT1*	Exostosin 1	Transmembrane glycoprotein er	Catalyzing the synthesis of heparan sulfate
*C1QTNF12*	Complement C1q tumor necrosis factor-related protein 12	Secreted protein extracellular	PI3K-Akt signaling pathway
*FBH1*	F-box protein, helicase, 18	Nucleus	Unwinds double-stranded DNA in a 3 to 5 direction
*FBXO34*	F-box protein 34	Nucleoplasm, cytoplasm	Protein-ubiquitin ligases
*GALNT6*	UDP-N-acetyl-alpha-D-galactosamine:polypeptide N-acetylgalactosaminyltransferase 6 (GalNAc-T6)	Golgi	O-linked glycosylation, GalNAc to serine and threonine residues on target proteins
*GDPD5*	Glycerophosphodiester phosphodiesterase domain containing 5	Plasma membrane? Cytosol?	Glycerol metabolism, osmotic regulation of cellular glycerophosphocholine
*GPR55*	G Protein-Coupled Receptor 55	Plasma membrane	Signaling pathway
*IL10RA*	Interleukin 10 receptor, alpha	Plasma membrane	Akt signalling, IL-10 receptor
*IL13*	Interleukin 13	Extracellular space – secreted	Interleukin 13
*KCNC3*	Potassium voltage-gated channel, Shaw-related subfamily, member 3	Plasma membrane	Voltage channel
*LMNA*	Lamin A/C	Nucleus/cytosol	Nuclear assembly, chromatin organization, nuclear membrane
*LRRC43*	Leucine rich repeat containing protein 43	Unknown	Unknown
*MAN1A1*	Mannosidase, alpha, class 1A, member 1	Golgi membrane/er/secreted?	Maturation of Asn-linked oligosaccharides
*MAPK8IP1*	Mitogen-activated protein kinase 8 interacting protein 1	Endoplasmic reticulum/mitocondria	MAPK signaling pathway and Akt Signaling
*MCART6 aka. SLC25A53*	Solute carrier family 25, member 53	Unknown	Unknown
*NCOR2*	Nuclear receptor corepressor 2	Nucleus	Transcriptional activity of SMAD2/SMAD3-SMAD4 heterotrimer
*NDFIP2*	Nedd4 family interacting protein 2	Endosome/golgi/ER	Interacts with HECT domain-containing E3 ubiquitin-protein ligases
*NPAS3*	Neuronal PAS domain protein 3	Nucleus	Transcription factor
*PDE4A*	Phosphodiesterase 4A, cAMP-specific	Plasma membrane/cytosol	cellular responses to extracellular signals
*PON1*	Paraoxonase 1	Extracellular space – secreted	Aryl-dialkyl phosphatase activity
*SH2B3*	SH2B adaptor protein 3	Cytosol	SH2B adaptor family of proteins, signaling activities by growth factor and cytokine receptors
*SLC9A7*	Solute carrier family 9, subfamily A (NHE7, cation proton antiporter 7), member 7	Endosome and Golgi	pH homeostasis in organelles along the secretory and endocytic pathways
*STIM1*	Stromal interaction molecule 1	Cytoskeleton, plasma membrane, ER	Mediates Ca^2+^ influx after depletion of intracellular Ca^2+^ stores by gating of store-operated Ca^2+^ influx channels (SOCs)
*TNRC6A*	Trinucleotide repeat containing 6A	Cytosol	Post-transcriptional gene silencing through the RNA interference (RNAi) and microRNA pathways
*TOX2*	TOX high mobility group box family member 2	Nucleus	Chromatin remodeling
*ZCCHC18*	Zinc finger, CCHC domain containing 18	Nucleus	Nucleic acid binding, TF
*ZNF365*	Zinc finger protein 365	Cytoplasm, centrosome	Regulation of mitosis?

**Figure 2:**
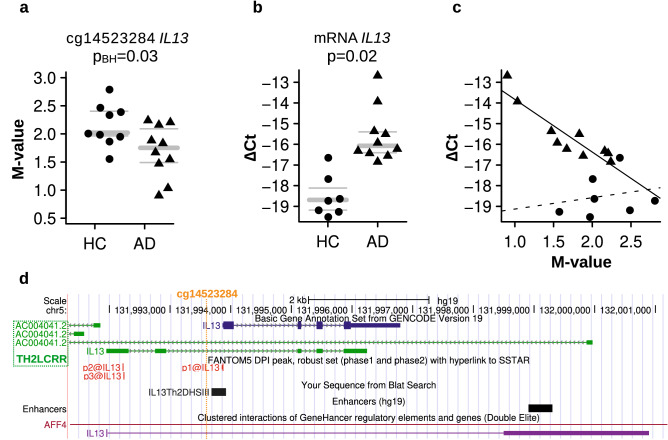
Differential DNA methylation and mRNA levels in the *IL13* gene in CD4^+^CLA^+^ T cells between AD patients and HC. (**a**) DNA methylation levels for the DMP located at the *IL13* promoter (cg14523284). Each dot represents an individual, HC (n = 9, circle) and AD patients (n = 10, triangle). P_BH_ = Benjamini Hochberg *p* value. (**b**) *IL13* mRNA levels in CD4^+^CLA^+^ T cells between HC (n = 7) and AD patients (n = 10) by qRT-PCR. Gray bars in the panels **a** and **b** indicate mean (bold), upper and lower (thin) quartiles. (**c**) Correlation between *IL13* mRNA levels and *IL13* DNA methylation levels. Lines of best fit are individually presented for AD (solid line, m < −2.5, *p* < 0.0005, R^2^ > 0.82) and HC (dashed line, m = 0.5, *p* = 0.65, R^2^ < 0.05). (**d**) Location of the CpG site cg14523284 at the promoter of *IL13* within the T helper type 2 locus control region associated RNA at Chr 5q31.1 (https://genome.ucsc.edu).

Some of the differentially methylated genes in CD4^+^CLA^+^ T cells of AD patients are implicated as key regulators of cytokine signaling pathways and immune inflammation (*ARHGEF3*, *ASB2, DAPP1, IL10RA*, *PDE4A*, *SH2B3, STIM1* and *TOX2*, Fig. 1c). We also found that CD4^+^CLA^+^ T cells from AD patients have significantly decreased methylation in the *IL13* promoter (Fig. 2). DNA methylation levels at the CpG site cg14523284 in the upstream region of *IL13* were significantly lower compared to the levels in HC (Fig. 2a**),** by contrast, mRNA levels for *IL13* were increased in AD patients **(**Fig. 2b**)**. Spearman correlation tests showed a significant inverse correlation between DNA methylation and *IL13* mRNA levels (Spearman rho −0.63, *p* = 0.006) (Fig. 2c), mainly driven by AD cases. This CpG site with reduced methylation levels in AD patients was at the proximal upstream of the Th2-specific DNA hypersensitive site in the *IL13* promoter but within the Th2 locus-control long non-coding RNA^37^ (Fig. 2d), indicating that this epigenetic modification might functionally explain the augmented capability of CD4^+^CLA^+^ T cells of AD patients to produce IL-13. Correlations computed within each group further strengthen the distinct AD vs HC responses, showing a clear trend within the former group (Spearman rho < −0.92, *p* < 0.0002, FDR < 0.003, n = 10) but no statistically significant relationship in the latter (Spearman rho = 0.39, *p* > 0.38, FDR > 0.38, n = 7). A linear regression to each of the two datasets shows a statistically significant relationship between delta-Ct and M-value in AD (*p* < 0.0005, R^2^ > 0.82) but not in HC (*p* > 0.65, R^2^ < 0.05) (Fig. 2c).

### miRNA deregulation in CD4^+^CLA^+^ T cells of AD patients

**Figure 3:**
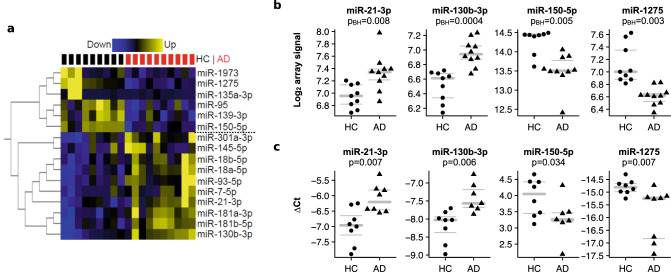
Differentially expressed miRNAs in CD4^+^CLA^+^ T cells between AD patients and HC. (**a**) Differential miRNA expression by miRNA microarray between HC (n = 9) and AD patients (n = 10). Fold expression of 16 miRNAs with significant differences between AD patients and HC (Benjamini Hochberg corrected *p* value < 0.05). Blue indicates downregulation and yellow indicates upregulation. Each row corresponds to a miRNA and each column to 1 sample. Black and red squares on the top indicate HC and AD samples, respectively. Six down-regulated and 10 up-regulated and miRNAs in AD patients are indicated to the right of the heatmap. Software used Glucore Omics Explorer (https://www.qlucore.com/). (**b**) Log_2_ miRNA levels from the microarray analysis between HC and AD patients. The array level indicates the amount of miRNA based on the fluorescence signal in the Cy3 channel. P_BH_ = Benjamini Hochberg *p* value. (**c**) ∆-Ct miRNA levels confirmed with qPCR between HC and AD patients. Gray bars in the panels B and C indicate mean (bold), upper and lower quartiles; each dot represents an individual.

**Figure 4:**
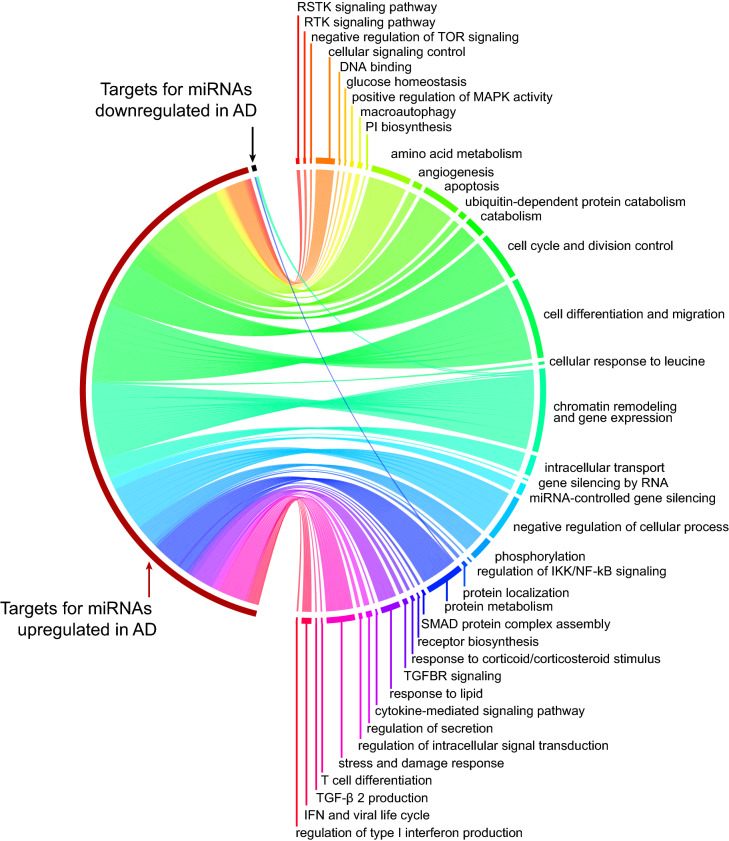
Functional enrichment analysis on the targets of AD perturbed miRNAs (FDR < 0.05). On the right hand key biological processes are summarized by their similarity. The lines are proportional to the number of biological processes associated with targets of miRNAs that are upregulated (red) or downregulated (black) in AD patients. The complete list of 202 processes is presented in Supplementary Table S2 online. This circular layout was created with the free R package circlize (https://cran.r-project.org/web/packages/circlize/index.html).

The analysis of global miRNA expression levels in the four T cell populations revealed that only the CD4^+^CLA^+^ T cells contain differentially expressed miRNAs (n = 16) between AD patients and HC (BH corrected *p* value < 0.05). In AD patients, 10 miRNAs were up-regulated, and 6 miRNAs were down-regulated, allowing a clear distinction between AD patients and HC (Fig. 3a). We selected 8 differentially expressed miRNAs from the microarray analysis (miR-7-5p, miR-21-3p, miR-93-5p, miR-130b-3p, miR-145-5p, miR-150-5p, miR-181b-5p and miR-1275) for technical validation by qPCR. Significant differences between AD patients and HC could be confirmed by qPCR for four of them, miR-21-3p, miR-130b-3p, miR-150-5p and miR-1275 (Fig. 3b,c). Next, we performed gene set enrichment analysis on the predicted miRNA targets of upregulated and downregulated miRNAs in AD (Fig. 4) and found 202 biological processes associated with the targets of the miRNAs dysregulated in AD (Supplementary Table S2 online). The top pathways (FDR < 1.1 × 10^−5^) included cell differentiation and migration, apoptosis ubiquitin-dependent protein catabolic process, transforming growth factor beta receptor signaling pathway and positive regulation of MAP kinase activity. We found that *ESR1*, *NDFIP2*, *ASB2* and *TNRC6A* genes which were differentially methylated in AD patients (Table 4) were also targeted by upregulated miRNAs in AD patients (Supplementary Table S2 online), suggesting complex interactions between these epigenetic layers.

### Network analysis of coordinated epigenetic responses reveals potentially perturbed pathways in circulating CD4^+^CLA^+^ T cells from AD patients

**Figure 5:**
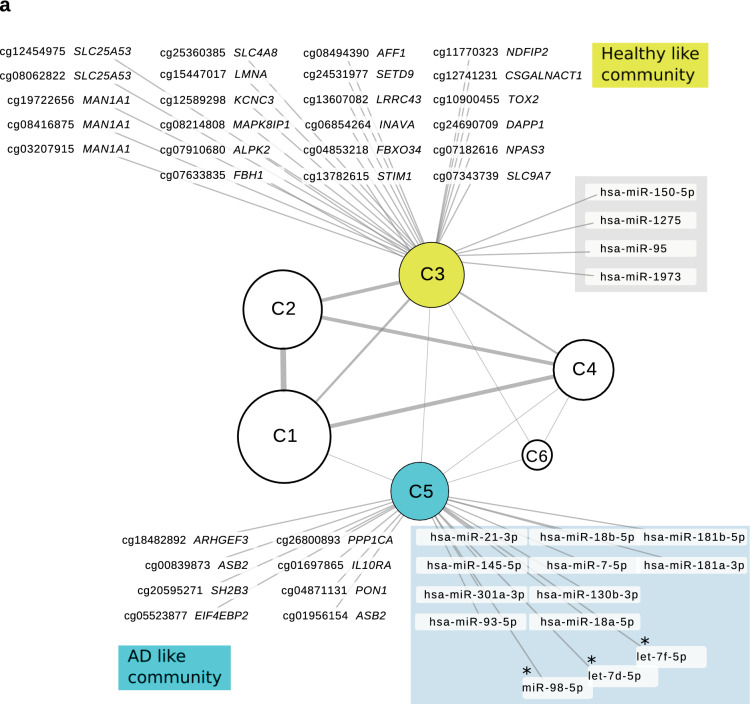

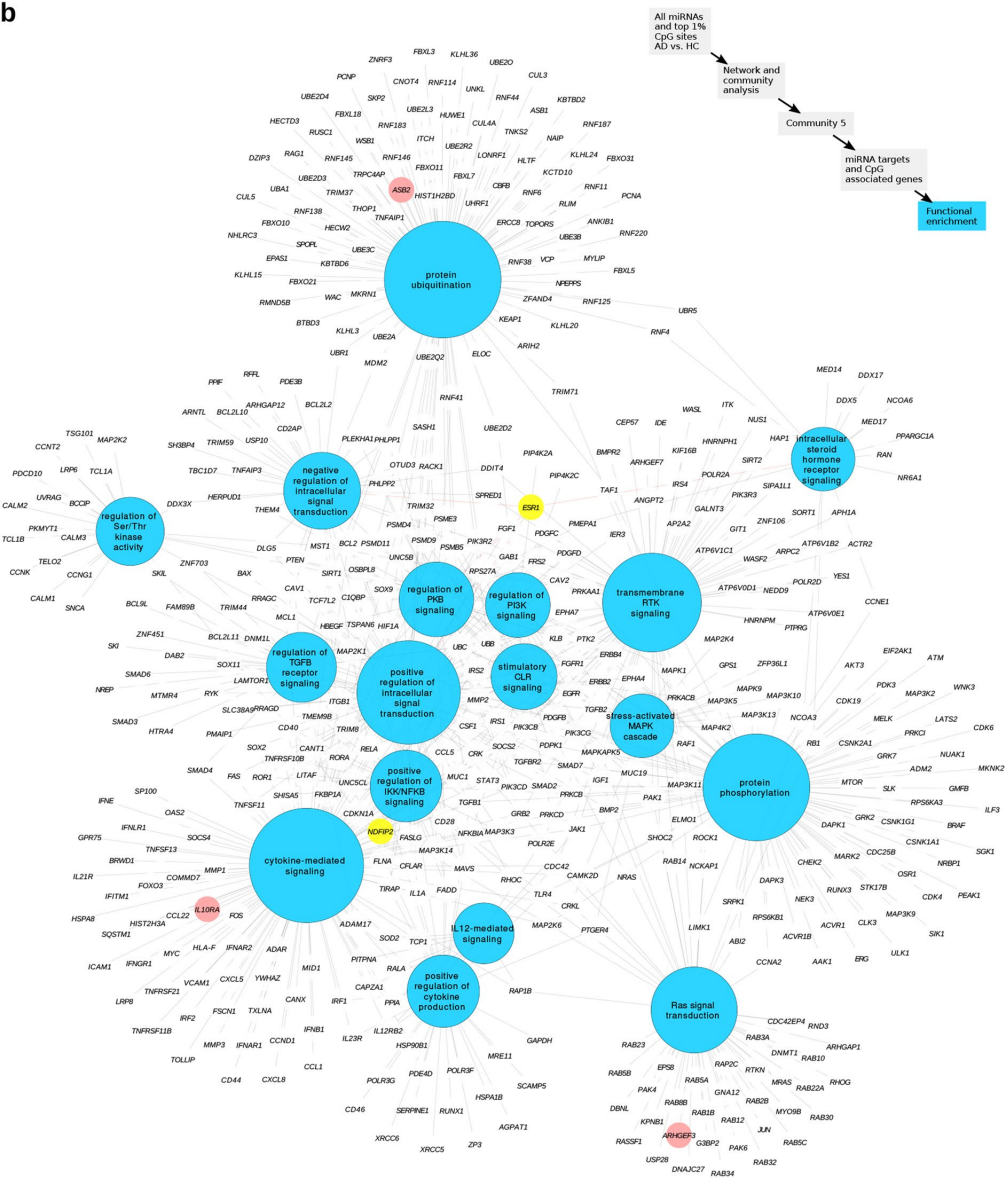
Integrated network analysis of miRNAs and CpG sites (CpGs) highlights coordinated epigenetic changes in CD4^+^CLA^+^ T cells. (**a**) Based on the set of associated miRNAs and CpG sites (absolute Spearman ρ > 0.75, FDR < 0.001) we identified 6 communities (C1–C6) of highly interconnected miRNAs/CpGs. These shows coordinated expression patterns, with strong interconnections within but not between communities. Note that communities 3 (yellow) and 5 (blue) show many of the differential miRNAs/CpGs detected in HC and AD patients: CpG sites (black), miRNAs (shadow), and miRNAs targeting *IL13* (with asterisk). The node size for each community is proportional to the number of elements in each community. Line thickness connecting the communities is proportional to the number of connections between them. Detailed information on these communities is presented in Supplementary Table S3 online. (**b**) Functional enrichment analysis on putative regulated targets revealed 17 key pathways associated with AD (community C5) and are indicated as blue circles. Genes highlighted in pink and yellow were also detected as differentially methylated in the comparison between AD patients and HC (Table 2). The networks were created in Cytoscape 3.7.2 (https://cytoscape.org/).

To further explore the coordinated epigenetic relationships, we performed network analysis using the full set of miRNAs, as well as the top ~ 1% CpG sites showing the largest differences between AD and HC. An association analysis (absolute Spearman *ρ* > 0.75, FDR < 0.001) was used to identify strongly interconnected communities of miRNA/CpG sites. Communities are sets of miRNA/CpG sites that tend to display globally coordinated expression patterns, thus highlighting potentially harmonized regulatory effectors^38^. Importantly, the set of miRNA/CpG sites within a community are more strongly associated with their respective community than between communities. Our analyses highlighted six communities based on the global coordinated associations between miRNA and CpG sites. Interestingly, we identified one miRNA/CpG community (C5) that recapitulated the genes showing significant differences in the AD group (Fig. 5a). This community has 640 elements: 122 miRNAs and 518 CpG sites that included 8 out of 12 CpG sites with increased DNA methylation in AD patients (Table 2) but none with decreased methylation. This community also contained all 10 miRNAs that had been identified as upregulated in the AD group by the differential expression analysis (Fig. 3a), suggesting that our approach was powered enough to capture AD associated features and its neighbors including three miRNAs targeting *IL13* (hsa-miR-98-5p, hsa-let-7d-5p, hsa-let-7f.-5p) (Fig. 5a). On the other hand, community C3 has 765 elements: 15 miRNAs and 765 CpG sites including CpG probes with increased DNA methylation in HC (*DAPP1*, *STIM1*, *PDEA4* and *TOX2*) and 4 miRNAs with increased expression in HC (Fig. 1b & Fig. 3). Overall, communities C3 and C5 reflect the antagonistic behavior that we had observed in HC and AD patients (Fig. 1b). Detailed information on the CpG sites and miRNAs detected in each community is presented in Supplementary Table S3 online.

The list of miRNAs targets together with the genes annotated to contain CpG sites associated with each community were further functionally characterized (Supplementary Table S4 online). Community 5 (C5) showed many processes related to cell signaling. Indeed, top pathways (FDR < 10^−8^) associated with community C5 revealed 17 involved in protein ubiquitination, positive regulation of intracellular signal transduction, protein phosphorylation, positive regulation of IKK/NFkB signaling, Ras protein signal transduction, stress-activated MAPK cascade and cytokine-mediated signaling (Fig. 5b). Overall, this suggests that epigenetic alterations in CD4^+^CLA^+^ T cells of AD patients may be affecting receptors and adaptors that are crucial for the regulation of cytokine signaling. Genes that showed increased DNA methylation in AD were represented in these pathways, and included *ASB2* in protein ubiquitination, *IL10RA* in cytokine mediated signaling and *ESR1* as a common gene in several C5 pathways (Fig. 5b).

## Discussion

Several studies have shown disturbed biology in skin homing CLA^+^ T cells in AD patients but the underlying mechanisms explaining the alterations in this cell population remain unclear. We here analyzed the combined profiles of DNA methylation and miRNA expression in sorted peripheral blood T cell populations from AD patients compared to HC. This study revealed for the first-time significant differences in the DNA methylation levels of several key immune genes in skin homing CD4^+^CLA^+^ T cells from AD patients. The most significant differences among the 40 differentially methylated genes were found in *ASB2*, *DAPP1*, *FBXO34* and *NDFIP2* **(**Table 2**)**. We also found significant DNA methylation differences in genes known to be genetically associated with AD predisposition including *IL13*^39,40^, *IL10RA*^41^, *ZNF365*^42^ and *STIM1*^43^.

Our results revealed for the first time a significant inverse correlation between reduced DNA methylation in the *IL13* promoter and increased *IL13* mRNA expression in CD4^+^CLA^+^ T cells of AD patients (Fig. 2c), providing insights into the molecular events that might lead to the remarkable ability of CLA^+^ T cells from AD patients to secrete IL-13^22,24,44^. The relationship between reduced DNA methylation in the *IL13* promoter of AD patients and increased IL-13 expression support that this cytokine is a central pathogenic mediator in AD^45^ and therapies targeting this molecule or its receptors (*e.g*. Dupilumab) might be beneficial in patients with severe atopic phenotypes^46^. The increased DNA methylation levels in the *IL13* promoter observed in HC could explain why the *IL13* mRNA expression was significantly reduced in this group (Fig. 2). DNA methylation differences in the *IL13* gene (cg04303330) has been also described by Boorgula et al., in whole blood samples from patients with the phenotype of AD with eczema herpeticum^47^.

We also found reduced methylation in the gene *PDE4A* in AD patients, a molecule that promotes downstream inflammatory pathways and is amenable to be targeted with phosphodiesterase inhibitors (Fig. 1b)^48^. Since CLA^+^ T cells recirculate between skin and blood and mediate pathogenic inflammation, antibodies targeting adhesion molecules used by CLA^+^ T cells to mediate their transendothelial migration^49^ or anti-CLA antibodies are being attempted and proposed as promising therapeutic options in AD^50^. However, more studies are needed because CLA^+^ T cells are involved in immunosurveillance and preventing their migration to skin produces CLA^+^ leukocytosis and possibly alterations in other tissues.

We discovered 16 miRNAs to be differently expressed in CD4^+^CLA^+^ T cells from AD patients (Fig. 3)**,** all except for miR-21 previously undescribed in AD^12^. The magnitude of the expression differences between patients and controls was small, but the effect sizes for miRNA variation are unknown. The up-regulation of miR-21 and miR-145 may reflect the pro-inflammatory status of CD4^+^ CLA^+^ T cells in AD patients. Indeed, miR-21 is involved in the polarization of adaptive immune responses and has been found upregulated in lesional skin biopsies of AD patients and in lesional skin of patients with contact dermatitis after challenge with diphenylcyclopropenone^51^. The altered miRNA signatures in AD may differ depending on the cell type or tissue studied. We did not find any differences in the expression of miR-146a or miR-155 between AD and HC, previously reported to be up-regulated in lesional skin biopsies of AD patients^52,53^ consistent with that in those studies the significant differences were attributed to the keratinocytes. Moreover, we discovered 202 biological processes significantly enriched in targets of AD-associated miRNAs of which many were implicated in cell signaling, transforming growth factor beta production and interferon responses (Fig. 4). These analyses highlighted *ESR1* as a target of several AD upregulated miRNAs, involved in several processes such as cell differentiation and migration, transforming growth factor beta receptor signaling pathway and T cell differentiation (Supplementary Table S2 online); suggesting for the first time that this transcription factor is a dysregulated mediator of several putative disturbed pathways in CLA^+^ T cells from AD patients. These observations are in line with recent studies showing that estrogen-responsive genes may influence IL-13 production in patients with eosinophilic esophagitis^54^. We also evaluated putative targets of the dysregulated miRNAs in CD4^+^CLA^+^ T cells, not only for genes expressed in CD4^+^ T cells but using the entire miRTarBase targets, since these altered miRNAs may be released from the T cell and exert their effects on skin cells, endothelium or other immune cells.

The integrative network analysis of coordinated changes of DNA methylation and miRNA in CD4^+^CLA^+^ T cells revealed several pathways and new candidate dysregulated genes for AD (Fig. 5b). These included *ASB2* a gene encoding the ankyrin-repeat suppressor of cytokine signaling (SOCS) box-containing protein 2, an E3 ubiquitin ligase that promotes protein degradation by coupling SOCS proteins with the elongin BC complex and mediate the ubiquitination of Notch targets such as E2A and Janus kinase (Jak) 2^55^ and Jak3^56^. ASB2 is known to be expressed in T helper 2 cells (Th2), is regulated by GATA3 and promotes Th2 phenotype *in vivo*^57^. Further studies are needed to elucidate the role of ASB2 in CLA^+^ T cells from AD patients. In contrast, HC showed increased methylation in *NDFIP2* a gene encoding for the Nedd4 Family Interacting Protein 2 which has been described to limit the cytokine signaling and expansion of effector Th2 cells^58^ by promoting degradation of JAK1, probably by ITCH- and NEDD4L-mediated ubiquitination^59^. Two other genes from the F-box family (*FBXO18* and *FBXO24*) implicated in protein ubiquitination were found differentially methylated in AD patients (Table 2), altogether suggesting that genes implicated in protein ubiquitination are epigenetically altered in CD4^+^CLA^+^ T cells from AD patients, and may promote inflammation by altering signaling. Ubiquitin ligases also mediate inhibition of activation of induced cell death (AICD) and contribute to lymphocyte accumulation^60^, therefore, altered epigenetic signatures in genes involved in protein ubiquitination may contribute to the resistance to apoptosis that acquires the CLA^+^ T cells upon skin infiltration^25^.

The coordinated epigenetic changes highlight several genes and pathways involved in intracellular signaling and were consistently associated with AD features in the CD4^+^CLA^+^ T cells from patients. These included cytokine-mediated signaling associated with *IL10RA* and its intracellular adaptor SH2B3 which constrains cytokine signals and might influence inflammatory immune responses in peripheral lymphoid tissues^61^. Also, the Ras signaling transduction pathway implicating *ARHGEF3*; as well as positive regulation of IKK/NFKB signaling implicating *NDFIP2* (Fig. 5b). Among genes in community C3, *TOX2* showed increased methylation in HC (Fig. 1b & Fig. 5a); this transcription factor is being recognized as a regulator of T cell differentiation but further studies are needed to evaluate its role in CLA^+^ T cells^62,63^.

We analyzed CD4^+^CD45RA^+^ naïve T cells since we hypothesized that epigenetic signatures predisposing to T cell dysregulation in AD patients may already be present at this cell stage before the effector or memory T helper cell differentiation. In agreement with Han et al.^64^, we found almost no differences in DNA methylation levels between CD4^+^ naïve T cells from AD patients compared to HC (Fig. 1a); neither in the unfractionated CD4^+^ T cells as described by Rodriguez et al.^11^, nor in CD8^+^ T cells supporting that epigenetic alterations are principally detected in CD4^+^CLA^+^ T cells. The CD8^+^CLA^+^ T cells also have an important role in AD^65,66^, although recent studies highlighted their pathogenic role in psoriasis^67^. Further studies are needed to evaluate epigenetic signatures in the CD8^+^CLA^+^ T cell populations in AD patients.

Provided that T cell turnover between skin and blood involves active de-homing from skin and migration through the lymph nodes and peripheral circulation^26^, it is conceivable that CD4^+^CLA^+^ T cells isolated from blood could be informative on pathogenic processes occurring in skin. It remains to be determined at which developmental state the CD4^+^CLA^+^ T cells acquired these altered epigenetic signatures in AD patients. Most probably by signals received after antigen priming or during their recirculation within skin, lymph nodes and the peripheral blood. Exposure to environmental signals from the skin microbiome or allergens could also be involved. We do not know the TCR specificity of the CD4^+^CLA^+^ T cells analyzed here but previous studies have shown that CLA^+^ T cells in AD preferentially respond to allergens and Staphylococcal enterotoxin B^68^.

**Figure 6:**
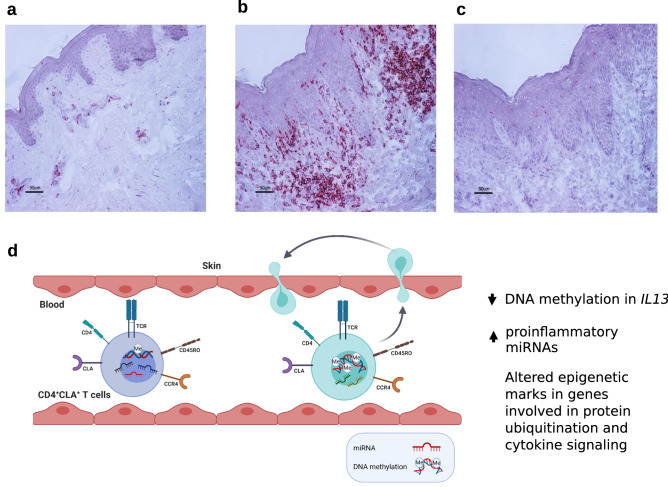
Identification of CLA^+^ cells in skin biopsies and epigenetic changes detected in circulating CD4^+^CLA^+^ T cells from AD patients that might contribute to skin inflammation. A representative immunohistochemistry staining of the distribution of CLA^+^ cells in skin biopsies from (**a**) a healthy control, (**b**) lesional skin from an AD patient, and (**c**), rat IgM used as isotype control. Scale bars represent 50 µm. (**d**) Circulating CD4^+^CLA^+^ T cells from AD patients (light blue cell) show significant differences in DNA methylation and miRNA levels compared to CD4^+^CLA^+^ T cells from HC (purple cell). The main differences were detected in the reduced DNA methylation of the *IL13* gene, the increased expression of proinflammatory miRNAs and coordinated epigenetic changes in genes involved in protein ubiquitination and cytokine signaling in AD patients. Since these CD4^+^CLA^+^ T cells can recirculate between skin and blood^25^, these altered epigenetic marks might contribute to AD immunopathology.

We did not compare frequencies of CLA^+^ T cells between AD patients and HC in peripheral circulation, however, in agreement with previous reports^25^, we found abundant infiltrates of CLA^+^ cells in the lesional skin biopsy specimens of the AD patients compared to HC **(**Fig. 6a-c)**,** which were also dominated by CD3^+^ and CD4^+^ cells in consecutive sections (data not shown). Given the larger numbers of these epigenetically altered CD4^+^CLA^+^ T cells in the skin of patients (Fig. 6b) and the recirculation of these cells, we hypothesize that the epigenetic alterations detected in CLA^+^ T cells from peripheral blood promote pro-inflammatory functions in skin of patients and contribute to AD immunopathology (Fig. 6d). It should be noted that AD is a heterogeneous disease with different immunophenotypes in the cellular infiltrates, including not only Th2 inflammation but also other cell populations^69–71^. We here included adult patients with a phenotype of severe AD (objective SCORAD above 40) and allergen specific IgE sensitization. Therefore, the epigenetic signatures detected in this study and their related genes may be associated with this phenotype of patients, and more research is needed to evaluate their implications in other AD cohorts.

Several methodological aspects add strength to this study. To avoid the confounding effects that cell heterogeneity has on the interpretation of epigenetic and expression studies, we analyzed sorted T cell populations in peripheral blood. In addition, we used a robust methodology that interrogated DNA methylation levels in ~ 450 000 CpG sites through the genome and global miRNA expression in ~ 2000 miRNAs. At several loci, the magnitude of the epigenetic differences between AD and HC were relatively small, yet statistically significant after correction by multiple testing. Several of the loci detected in the comparison between AD patients and HC (Fig. 1b & Table 2) showed coordinated changes when analyzed with a different algorithm for network analysis (Fig. 5a). Our study has also some limitations, to avoid the confounding effects of gender, we included only male AD patients that even though they were selected to be as much homogeneous as possible had some differences in their total serum IgE levels, AD severity and the presence of comorbidities such as asthma (Table 1). It is worth noting that a larger sample size would assist in identifying important biological signals in the etiology of AD. Due to the limitations in the amount of cells that were sorted per individual, we could only measure mRNA expression in few genes by TaqMan. We detected significant differences in *IL13* expression, but we could not interrogate how the coordinated changes in miRNA and CpG methylation affects global gene expression. Nevertheless, this study revealed new genes and biological processes that deserve to be further validated at mRNA and protein levels in other cohorts of AD patients and functionally evaluated at the cellular level to dissect their role in AD pathogenesis.

In summary, we here discovered putative altered molecular pathways in circulating CD4^+^CLA^+^ T cells from AD patients, involving disease-associated signatures in DNA methylation and miRNA levels. The identified loci highlight new candidates in AD, including genes mediating intracellular cell signaling and adaptor molecules of the IL-10/IL-13 interleukin signaling pathway as well as genes involved in protein ubiquitination. Our findings support epigenetic profiling as a valuable tool to uncover putative molecules participating in disease pathways. Further studies are needed to define the downstream effects of these epigenetic alterations in AD immunopathology and evaluate if environmental signals at the target organ (*e.g*., skin microbiota) induce the detected epigenetic differences in circulating CD4^+^CLA^+^ T cells from AD patients.

## Methods

### Study population and samples

Twenty age-matched adult male individuals (10 AD patients and 10 HC) were selected from a Swedish eczema study^72^. All participants were asked to visit the Dermatology Unit at the Karolinska University Hospital in Solna and were examined by a dermatologist for their general physical conditions, in case of AD patients, also for the severity of the eczema. Afterwards, all provided a 450 ml blood donation (see below). The cases fulfilled the following inclusion criteria: (1) a physician-diagnosis of moderate or severe AD as determined by the objective Scoring Atopic Dermatitis index (SCORAD) (2) elevated levels of total plasma IgE > 122 kU_A_/L (ImmunoCAP, Thermo Fisher Scientific, Uppsala, Sweden), (3) positive allergen-specific IgE > 0.35 kU_A_/L as determined by Phadiatop (Thermo Fisher, Uppsala, Sweden), and (4) besides AD, a clinical history of asthma and/or rhinitis. All AD patients had used topical corticosteroids at least 30 days before blood sampling. Controls were healthy individuals without clinical history of eczema, asthma or other allergic diseases and plasma IgE levels below 122 kU_A_/L. On the same day as the 450 ml blood donation, skin biopsies were obtained from AD patients (n = 5) and from HC (n = 5). Punch biopsies (4 mm) were taken under local anesthesia (Lidocaine 5 mg/ml with epinephrine 5 µg/mL, Astra, Södertälje, Sweden) from eczema lesions in the AD patients located in the popliteal fossa, upper/mid back or shoulders and healthy skin of HC from the low-back (lumbar) region. The biopsies were snap-frozen on dry ice and stored at −80 °C until immunohistochemistry analysis (see below). This study was conducted in accordance with the Helsinki Declaration ethical principles for medical research and was approved by the Regional Ethical Review Board in Stockholm (Dnrs 04–593/1, 2008/1122–32, 2010/754–32, and 2011/1051–31). All participants gave their written informed consent.

### Isolation of T cell populations

A 450 ml blood donation was obtained at the Blood Transfusion Center Karolinska University Hospital and processed immediately for cell isolation. Peripheral blood mononuclear cells (PBMCs) were separated by density centrifugation on Ficoll-Paque Plus (GE Healthcare, Uppsala, Sweden) and then labeled for magnetic associated cell sorting (MACS) to obtain CD4^+^ T cells and CD8^+^ T cells, CD4^+^CD45RA^+^ naïve T cells and skin-homing CD4^+^CLA^+^ T cells (Miltenyi Biotec, Gladbach, Germany). Cell viability after sorting was > 90% in all populations (as determined by trypan blue exclusion) and the purity was examined by flow cytometry (see below). Two aliquots were stored at −80 °C: one containing 5 × 10^6^ cells for DNA extraction as described previously^36^ and another containing a maximum of 10 × 10^6^ cells homogenized in QIAzol lysis reagent (Qiagen, Hilden, Germany) for total RNA extraction.

### Flow cytometry

Cells were re-suspended in FACS buffer (0.1% BSA in PBS) at a final concentration of 0.1 × 10^6^ cells per tube. Fc-receptors were blocked with 1 µl of normal mouse serum (Dako A/S, Glostrup, Denmark) for 10 min at 4 °C. Optimized panels of fluorochrome-conjugated monoclonal antibodies (Supplementary Table S1 online) were added to the cells, in a final volume of 100 µl and incubated for 30 min at 4 °C. Every staining included the unstained sample and the corresponding panels of isotype controls to set the gates for positive and negative populations. In addition to the markers described in Supplementary Table S1 online, which were measured in all the sorted samples, we also analyzed the cell surface expression of CCR4 (HC = 1, AD = 1) to better define the immunophenotype of the CD4^+^CLA^+^ T cells during validation of the cell sorting protocol (PerCP Anti-human CD194 (CCR4), clone L291H4, mIgG1 κ. Biolegend). Data were acquired using a FACS Calibur (BD Biosciences, San Jose, CA, USA), to at least 5000 events per population and analyzed by FlowJo vX.0.7 (FlowJo, LLC, Ashland, OR, USA).

### DNA methylation analysis

Genomic DNA was extracted using the QIAamp DNA Mini kit (Qiagen) and DNA concentrations were assessed by Qubit and Nanodrop. DNA purity was evaluated by the A260/A280 ratio by Nanodrop. After diluting to normalize the concentrations the DNA samples were bisulfite treated using the EZ-96 DNA Methylation kit (Zymo Research Corp., Irvine, CA, USA) according to manufacturer’s instructions. Denatured bisulfite-treated DNA was amplified, fragmented and hybridized onto the Infinium HumanMethylation450 BeadChip (Illumina Inc., San Diego, CA, USA), following manufacturer’s instructions, at the Mutation Analysis Core Facility (Karolinska Institutet, Stockholm, Sweden). The *.idat* files for each sample obtained from Genome Studio were imported in R and read in using the *minfi* package^73^ with the genome annotation based on GRCh37/hg19. A total of 77 samples were analyzed: 20 CD4^+^(HC = 10, AD = 10), 18 CD4^+^CD45RA^+^ naïve (HC = 8, AD = 10), 19 CD4^+^CLA^+^ cells (HC = 9, AD = 10), and 20 CD8^+^ (HC = 10, AD = 10). The data were normalized using the subset-quantile within array normalization (Swan) method^74^. The probes overlapping with known SNPs were removed, leaving 398 494 CpG sites for further analysis. Differentially methylated CpG sites between AD patients and controls were obtained using linear models (*y* ~ group + subject + age) and pairwise comparisons with empirical Bayes as implemented in *limma*^75^. DMRs were analyzed with the *minfi* package^73^. All the CpG sites presented as having differential methylation between AD patients and HC had a *p* value < 0.05 after Benjamini-Hochberg (BH) correction for multiple testing.

### RNA isolation and miRNA measurements

mRNA and miRNA were obtained by phenol/chloroform extraction. RNA integrity was evaluated using the Nanochip kits in Bioanalyzer (Agilent Technologies, Santa Clara, CA, USA) resulting in RIN values (mean ± SD) of 9.31 ± 0.38. miRNA levels were measured for 4774 probes (representing 2006 human miRNAs, miRBase database release 19) using the SurePrint G3 (8 × 60 K) miRNA array (Agilent Technologies). The raw *.txt* files obtained from the Agilent feature extraction software were imported in R and analyzed with *limma*^75^. After quality check 71 samples remained for the analysis of differential miRNA expression: 17 CD4^+^ (HC = 8, AD = 9), 15 CD4^+^CD45RA^+^ naïve (HC = 6, AD = 9), 19 CD4^+^CLA^+^ (HC = 9, AD = 10) and 20 CD8^+^ (HC = 10, AD = 10). To ensure homogeneity of analytical strategy with the DNA methylation, the same statistical approach described above was also used to find differentially expressed miRNAs. Experimentally validated human miRNA targets were determined through Ingenuity Pathway Analysis (IPA) (https://www.ingenuity.com) and miRTarBase^76^.

### Network analysis

Association analyses were performed after removing features with null variance and based on the entire set of quantified miRNAs and a subset of DNA methylation probes as follows. The full DNA methylation set comprising > 398,000 probes was filtered by first determining CpG probe UCSC classifications for the Illumina 450 K manifest through the FDb.InfiniumMethylation.hg19 R package in R 3.6.1, and excluded if no gene association was identified (e.g. intergenic probes). Secondly, we selected the top varying CpG probes (FDR < 0.1, 1.44% top probes) based on the *limma* comparison of AD patients and HC. Association analyses were then performed on the resulting set of 455 miRNA and 4369 CpG probes by Spearman correlations. *P* values were corrected for multiple hypothesis testing considering Benjamin-Hochberg false discovery rates as indicated throughout. The resulting set of positively correlated interactions were processed through igraph (https://igraph.org/) in Python 3.7. Node communities were computed through the Leiden algorithm through modularity optimization^38^ and antagonistic feature relationships were identified as described by Benfeitas *et al*^77^.

### Functional annotation

Gene set enrichment analysis was performed for Gene Ontology Biological Processes (2018) and KEGG 2019 pathways through the Enrichr^78^ API in Python 3.7 and considering the entire set of possible miRNA target genes and CpG-associated genes as background, where applicable. GO terms were further filtered for redundancy based on their similarity through Revigo^79^. Associations with miRNA targets were plotted using circus through the R circlize package^80^ and Cytoscape 3.7.2 (https://cytoscape.org/).

### qPCR validation of miRNA and mRNA expression in CD4^+^CLA^+^ T cells

To validate differentially expressed miRNAs, cDNA was prepared from the total RNA of CD4^+^CLA^+^ T cells (15 ng) from 10 AD patients and 9 HC (the same samples as used in the Agilent array) using the miRCURY LNA Universal RT microRNA PCR kit (Exiqon A/S, Vedbaek, Denmark) according to the manufacturer’s protocol. miRNAs were amplified using the miRCURY LNA SYBR Green master mix and specific PCR primer sets for 8 miRNAs (Exiqon) according to the manufacturer’s instructions. *SNORD44* (snRNA) was used as endogenous control as described previously^81^. To validate mRNA targets, cDNA was prepared using the high capacity RNA to cDNA kit (Life Technologies) starting from 80 ng of total RNA from 8 HC and 10 AD patients. Gene expression levels (mRNA) were measured using validated Taqman probes (Applied Biosystems, Foster City, CA, USA) according to manufacturer’s instructions using beta-2 microglobulin (*B2M*) as the reference gene. Quantitative real-time PCR was performed in the Biorad CFX96 system (BioRad Inc., Hercules, CA, USA) with each sample in duplicate, and the gene expression data were exported from the DataAssist Software v3.01. Differential expression between AD patients and HC was calculated using the comparative Ct method. In brief, the average Ct value for each miRNA was subtracted from the Ct value of *SNORD44* to obtain the delta-Ct. The comparisons of delta-Ct between AD patients and HC were calculated by unpaired *t*-tests and a *p* value < 0.05 was considered significant.

### Immunohistochemistry

The frozen skin biopsy specimens were embedded in optimal cutting temperature compound and six µm thin sections were prepared in a microtome-cryostat, two sections per glass slide. The tissue architecture and degree of cell infiltrates were evaluated by hematoxylin and eosin staining. For the evaluation of T cell markers, the sections were acetone fixed and stained using the avidin–biotin complex method (Vectastain Elite ABC-kit, Vector Laboratories, Burlingame, CA, USA) according to the manufacturer’s instructions with the following primary antibodies: anti-CLA (rat IgM, clone: HECA-452), anti-CD4 (mouse IgG_1_, clone: SK3), anti-CD3 (mouse IgG_1_, clone: SK7), all from BD Pharmingen (San Diego, CA, USA). Rat IgM (clone R4-22, BD Pharmingen) and mouse IgG_1_ (clone MG1-45, Biolegend) were included in each staining as isotype controls. Biotinylated antibodies targeting rat IgM (clone G53-238, BD Pharmingen) and mouse IgG_1_ (BA-2001, Vector Laboratories) were used as secondary antibodies. The sections were counterstained with hematoxylin.

### Statistical analysis

Statistical approaches were chosen according to the diverse data types in this study and explained in their respective methods sections. Hypothesis testing was performed by considering the null hypothesis of the absence of an association between the compared variables. The associations were tested according to the nature of the data: continuous vs continuous (Spearman rank correlation test); continuous vs categorical (*t*-test or Mann Whitney test according to data distribution). *p* values were corrected by the Benjamini–Hochberg procedure for multiple testing and a value < 0.05 was considered statistically significant.

## Supplementary information

Supplementary file 1

Supplementary file 2

Supplementary file 3

Supplementary file 4

## Data Availability

The authors declare that data supporting the findings of this study are available within the paper and its supplementary information files. DNA methylation and miRNA levels are available from the corresponding authors on reasonable request.
