# Supplementary material for: Epigenetic alterations in skin homing CD4+CLA+ T cells of atopic dermatitis patients
Source: Sci Rep. 2020 Oct 22;10:18020. doi: 10.1038/s41598-020-74798-z (PMC7582180; doi:10.1038/s41598-020-74798-z)
Supplement: Supplementary file 1 [file 41598_2020_74798_MOESM1_ESM.docx]

**Epigenetic alterations in skin homing CD4^+^CLA^+^ T cells of atopic dermatitis patients**

Nathalie Acevedo^1,2^, Rui Benfeitas^3^, Shintaro Katayama^4^, Sören Bruhn^5^, Anna Andersson^5^_,_ Gustav Wikberg ^6^, Lena Lundeberg ^6^, Jessica M. Lindvall^3^, Dario Greco^7,8,9^, Juha Kere^4^,

Cilla Söderhäll^4,10^ & Annika Scheynius^1,11^

^1^Department of Clinical Science and Education, Karolinska Institutet, and Sachs' Children and Youth Hospital, Södersjukhuset, Stockholm, Sweden;

^2^Institute for Immunological Research, University of Cartagena, Cartagena, Colombia;

^3^National Bioinformatics Infrastructure Sweden (NBIS), Science for Life Laboratory, Department of Biochemistry and Biophysics, Stockholm University, S-10691 Stockholm, Sweden;

^4^Department of Biosciences and Nutrition, Karolinska Institutet, Stockholm, Sweden;

^5^Department of Medicine Solna, Translational Immunology Unit, Karolinska Institutet, Stockholm, Sweden;

^6^Dermatology and Venereology Unit, Karolinska University Hospital, Stockholm, Sweden;

^7^Faculty of Medicine and Health Technology, Tampere University, Finland;

^8^Institute of Biosciences and Medical Technologies (BioMediTech), Tampere, University, Finland;

^9^Institute of Biotechnology, University of Helsinki, Finland;

^10^Department of Women´s and Children´s Health, Karolinska Institutet, Stockholm, Sweden;

^11^Science for Life Laboratory, Karolinska Institutet, Stockholm, Sweden

**Supplementary Table S1**. Cell surface expression of lineage markers in harvested cells as determined by flow cytometry.

| CD marker used for cell sorting | Number of samples  (HC/AD) | % of positive cells in HC (mean±SD) | % of positive cells in AD patients (mean±SD) | Antibody panel |
| --- | --- | --- | --- | --- |
| CD4^+^ | 10/10 | CD4^+^  (93.5 ± 6.3)  CD4^+^CD3^+^  (92.3 ± 2.8 ) | CD4^+^  (95.9 ± 0.7)  CD4^+^CD3^+^  (92.1 ± 1.3 ) | FITC α-human-CD14 (BioLegend, clone HCD14, mIgG_1_ κ).  PE α-human CD4 (BD, clone SK3, mIgG_1_ κ).  APC α-human CD3 (BioLegend, clone UCHT1, mIgG_1_ κ). |
| CD4^+^ CD45RA^+^ naïve | 7/8 | CD4^+^ CD45RA^+^  (87.7 ± 11.8 ) | CD4^+^ CD45RA^+^  (93.2 ± 2.3) | PE α-human CD4 (BD, clone SK3, mIgG_1_ κ).  PerCP/Cy5.5 α-human CD3 (BioLegend, clone UCHT1, mIgG_1_ κ).  APC α-human CD45RA (BD, clone H1100, mIgG_2b_ κ). |
| CD4^+^CLA^+^ | 9/10 | CLA^+^  (85.8 ± 9.5)  CD45RO^+^  (85.4 ± 9.2 ) | CLA^+^  (82.7 ± 4.8)  CD45RO^+^  (85.9 ± 7.2) | PE α-human CLA (Miltenyi Biotec, clone HECA-452, rat IgM).  PerCP/Cy5.5 α-human CD3 (BioLegend, clone UCHT1, mIgG_1_ κ).  APC α-human CD45RO (BD, clone UCHL1, mIgG_2a_ κ). |
| CD8^+^ | 10/10 | CD8^+^  (94.9 ± 5.6 )  CD8^+^CD3^+^  (89.7 ± 6.2) | CD8^+^  (95.7 ± 3.1)  CD8^+^CD3^+^  (88.3 ± 6.0) | FITC α-human CD8 (BD, clone HIT8a, mIgG_1_ κ).  APC α-human CD3 (BioLegend, clone UCHT1, mIgG_1_ κ). |
